# Maxillary Impacted Canine and Upper Lateral Incisor Agenesis Treatment with “Canine First Technique” and Clear Aligners: A Case Report

**DOI:** 10.3390/healthcare11162345

**Published:** 2023-08-20

**Authors:** Tecla Bocchino, Stefano Martina, Carolina Sangiuolo, Marzio Galdi, Alessandra Valletta, Vincenzo D’Antò

**Affiliations:** 1 Department of Neurosciences, Reproductive Sciences and Oral Sciences, University of Naples Federico II, Via Pansini 5, 80131 Naples, Italy; tecla.bocchino2011@gmail.com (T.B.); carosangiuolo@gmail.com (C.S.); marzio.galdi@gmail.com (M.G.); alessandra.valletta@unina.it (A.V.); vincenzo.danto@unina.it (V.D.); 2 Department of Medicine, Surgery and Dentistry “Scuola Medica Salernitana”, University of Salerno, Via Allende, 84081 Baronissi, Italy

**Keywords:** orthodontics, maxillary impacted canine, camouflage, extractions, clear aligners

## Abstract

The aim of this study was to show a case of an impacted canine in an adult patient with agenesis of the maxillary lateral incisor treated with clear aligners (CA). A 19-year-old male with a persistence of 5.3 and absence of 1.2 came to our department of the School of Orthodontics at the University of Federico II in Naples and asked for an aesthetic treatment. The Canine First approach was used to surgically expose the canine and pull it into the dental arch. In order to ensure long-term aesthetic, periodontal, and occlusal results, a treatment with CAs to close the space through the mesial placement of the canine and the enameloplasty of the tooth crown was performed. At the end of the treatment, the occlusal objectives were achieved.

## 1. Introduction

Nowadays, clear aligners perform an ever-increasing number of treatments, due to better aesthetic and comfort compared to braces [1], and they are a great technological development [2]. However, some complex malocclusions still represent a big challenge, especially when their correction includes movements that are difficult to achieve with aligners such as derotations of cuspids and bicuspids, and root torque [3,4].

Maxillary canine impaction has a prevalence of approximately 7% among Italian subjects [5] and in 85% of cases the impacted canine is palatal [6]. To evaluate the level of difficulty of treating a maxillary impacted canine, according to Ericson and Kurol, the alpha angle formed between the long axis of the canine and the interincisive midline and the position of the canine cusp in relation to sectors 1–5 are widely used [7]. Two theories have been proposed to explain the etiology of palatally impacted canines: the guidance theory described by Becker et al. in 1981 and the genetic theory described by Peck et al. in 1994 [8]. According to genetic theory, canine impaction could depend on the position of the canine germ, but over the years, it has been observed that the environmental component is predominant in the etiology of inclusions. However, genetics may influence the maxillary canine impaction in cases of dental anomalies in the upper lateral incisor [9]. Indeed, the guidance theory suggests that the distal aspect of the lateral incisor is the guide for canine eruption. Palatally impacted canines are very closely associated with lateral incisors that are peg-shaped, of small mesiodistal width, or congenitally absent [10].

One of the most common developmental anomalies is the absence of the maxillary lateral incisor (agenesis) [5]. In general, either the closure of the space, often with canine replacement, or the opening of the space for subsequent prosthetic restoration are the most widely used options to treat patients with congenitally missing maxillary lateral incisors [11,12]. Closing the missing lateral incisor space by lateralization of the canine and reshaping it to resemble the lateral incisor is preferable when comparing the above treatment options. This option is less expensive and less invasive. In addition, tooth-supported dental prostheses for the agenesis of the maxillary lateral incisor have worse scores in the periodontal indexes, whereas the closure of the space is evaluated as more aesthetically pleasing than the prosthetic replacement [13]. Several factors should be taken into account when designing a treatment plan to decide between these two options. These factors include the canine size, shape, and colour, location, patient age, patient profile, smile line, arch length tooth size discrepancy, ridge thickness, existing occlusion, and patient expectations of treatment [11]. Furthermore, to determine the level of commitment and motivation, the clinician should assess the patient’s psychological and behavioural profile [14]. In recent years, a growing number of orthodontics patients have been seeking “invisible” or “aesthetic” treatment with clear aligners; with these appliances bodily movement of the teeth can be difficult to achieve when the space closure is required to correct the congenital absence of one or both maxillary incisors [3]. Bodily movement of as much as 2.5 mm has been reported in cases of distalization, but this requires a high level of patient compliance [15,16]. There are no studies in the literature that describe the management of cases with impacted maxillary canines and agenesis of the maxillary lateral incisors with clear aligners.

The aim of the study is therefore to describe a case of a maxillary impacted canine and agenesis of the lateral incisor treated with a combined bracketless approach using the *Canine First Technique* and clear aligners.

The *Canine First Technique* consists of an innovative surgical–orthodontic approach for the impacted canine. This procedure provides the disimpaction of the canine, moving the crown away from the roots of adjacent incisors with a cantilever and a skeletal anchorage [17].

## 2. Case Report

### 2.1. Patient Information

In January 2019, a 19-year-old male patient came to the department of the School of Orthodontics at the University of Federico II in Naples with the main complaint being the persistence of a deciduous canine and the lack of eruption of the upper right lateral incisor.

### 2.2. Clinical Findings

An extraoral examination showed an oval and symmetric face, normal proportion of the facial third, poor dental exposure to smile, orthognathic profile, and a retruded position of the upper anterior limit of dentition.

**Figure 1:**
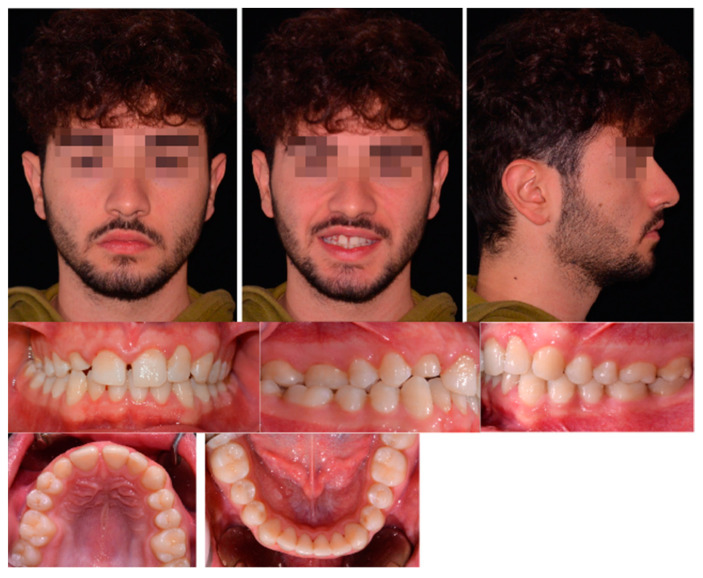
Pretreatment extra- and intra-oral records.

Intraoral examination and analysis of dental casts revealed a Class II subdivision malocclusion with a Class II molar relationship on the right side and a Class I relationship on the left side. The canine class relationship on the right side was not evaluable due to the persistence in the arch of 5.3. Cross-bite of the right posterior teeth was present (1.6–4.6, 1.7–4.7). The overjet was reduced, and the overbite was slightly increased. The upper incisor midline was slightly deviated to the right from the face midline and centered with the lower incisor midline. This shift of the upper midline was related to an absence of 1.2 and the persistence of the deciduous canine (5.3) in the upper arch. The periodontal health of the patient was good, with no bleeding on probing nor any sites with >3 mm of probing depth. All extra- and intra-oral records are shown in Figure 1.

The panoramic radiograph revealed an impacted maxillary canine and the agenesis of the upper right lateral incisor. The impacted canine was in sector 5 and had an alpha angle of 59° according to the Ericson and Kurol assessment [7]. Even 4.8 was not visible from the orthopantomography, while 2.8 and 3.8 were in eruption. The overall alveolar bone level was within normal limits.

**Figure 2:**
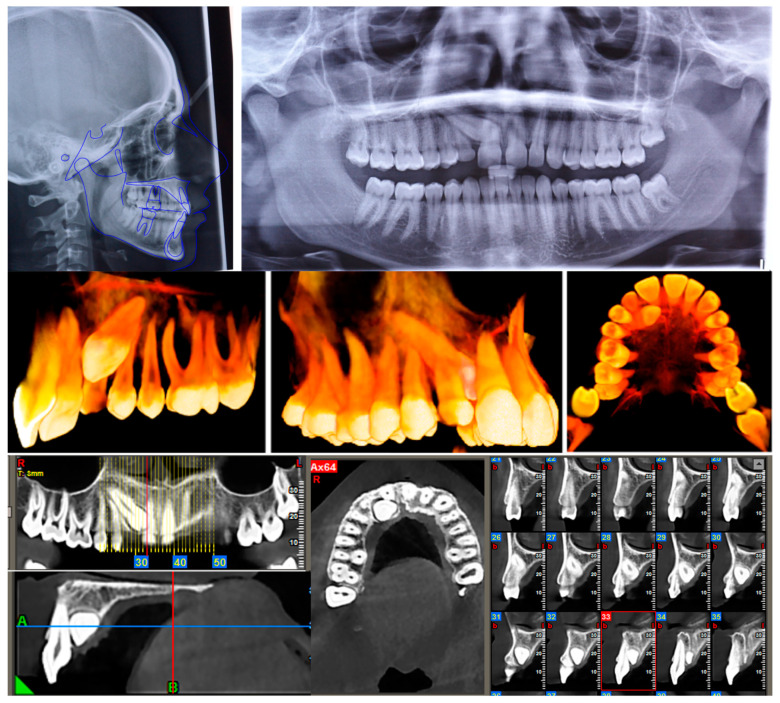
Pretreatment OPT, cephalometric analysis, and CBCT.

The cephalometric analysis indicated a skeletal class I, normodivergent growth pattern and normal position of the lower incisor (Table 1). Radiographic examinations are shown in Figure 2.

We prescribed a CBCT to evaluate the tridimensional position of the impacted canine and to better observe its relation with the roots of the other erupted teeth (Figure 2).

### 2.3. Diagnostic Assessment

The patient was diagnosed with a Class II subdivision right malocclusion with an agenesis of 1.2 and inclusion of 1.3.

The main treatment objectives were to recover the included canine, and transform it into a lateral, achieve a class I canine relationship with the premolar (that substitutes the canine) leaving the right molar in a class II relationship, and correct the cross-bite between 1.6 and 1.7.

The *Canine First Technique* was chosen to surgically expose and pull the canine. Next, multiple treatment options were presented to the patient: the use of orthodontic therapy to bring the canine back into the dental arch and close the space, or orthodontic repositioning of the canine, preservation of the agenetic lateral incisor space, and the following implant placement. The last hypothesis was rejected because of the patient’s young age and the unpredictability of the upper distalization, which exceeded 2.5 mm. Because the patient was seeking aesthetic therapy, treatment with fixed orthodontics was discarded and hybrid treatment with aligners was chosen.

### 2.4. Therapeutic Interventions

The patient underwent surgery to recover 1.3, in line with the *Canine First* approach [17]. Therefore, the 1.3 was surgically exposed by setting up a full-thickness flap and placement of a chain button on the palatal side; then, the flap was sutured, and the traction chain tied to the canine came out from the center of the flap.

At the same time as the surgical procedure, a miniscrew type 11 mm in length, 1.8 mm in diameter (Firma Plus—Sweden & Martina S.p.A., Due Carrare, Italy) was placed between the upper second premolar and the first molar on the right palatal side. A 0.019 × 0.025 TMA cantilever, with a force of about 100 g, was engaged in the slot of the miniscrew and used to pull the canine with a distalization and extrusion motion.

**Figure 3:**
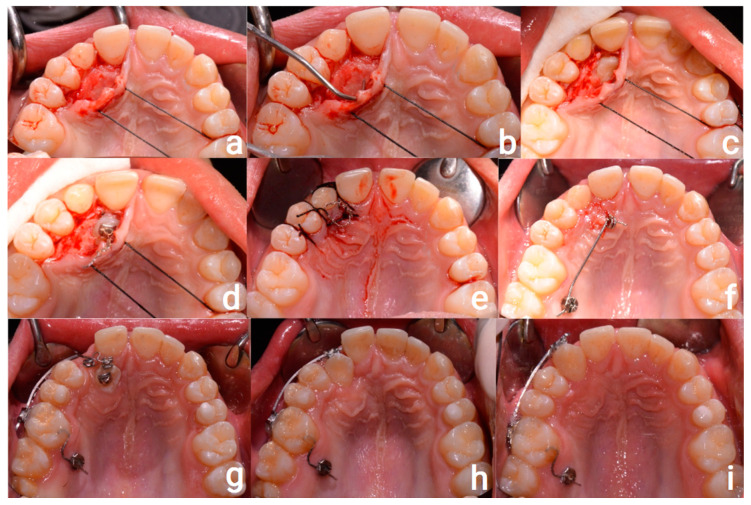
Operative sequence: (**a**) full-thickness flap preparation; (**b**) bone tissue removal; (**c**) canine exposure; (**d**) placement of the chain button; (**e**) suture; (**f**) TAD and 0.019 × 0.025 TMA cantilever placement; (**g**) buccal 0.019 × 0.025 TMA cantilever activated in vestibularization and distalization; (**h**) end of canine pull; (**i**) improvement of canine torque with a stainless steel wire).

The canine erupted 3 months after surgery; hence, it was slowly tractioned in the dental arch with Alastik from 1.3 to 5.3. Composite rampings were placed on 1.5 and 2.5 to allow the canine to have the correct position in the dental arch. After 4 months, 5.3 was extracted and a bracket was placed on 1.6, in which a 0.019 × 0.025 TMA cantilever was engaged and activated in vestibularization and distalization to complete canine pulling. Canine torque was improved using a sectional stainless steel wire and a 3.5 bracket (torque prescription: −17). The operating sequence is shown in Figure 3.

**Figure 4:**
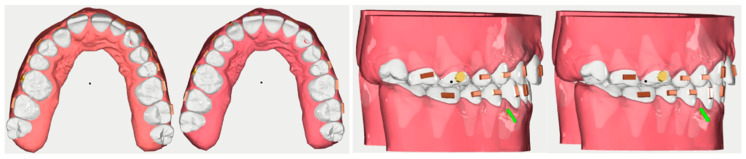
Initial and final steps of the virtual treatment plan in occlusal and lateral view.

At the end of this stage, a digital scan of both upper and lower arches was taken for prescription and fabrication of the aligners (UAB Ordoline, Vilnius, Lithuania). Sequential mesialization of the premolars and molars was planned, allowing for the maximum retentive surface contact of the aligners with the teeth being moved (Figure 4).

**Figure 5:**
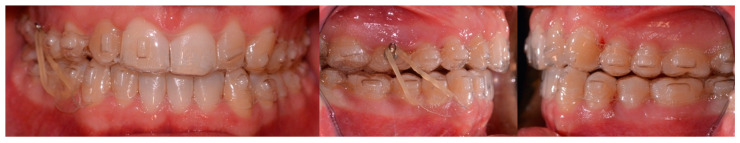
Class III elastics on the right side to mesialize canine and latero-posterior group.

Then aligners were used to close the space of the upper lateral incisor agenesis for mesialization of the canine. Attachments and IPR were performed at the delivery of the aligner number 1. Twenty-four aligners were used, for a total of seven months of treatment. The aligners were changed every 7 days. Class III elastics were used on the right side to mesialize the canine and latero-posterior group. No refinement stage was needed (Figure 5).

A lower retainer (L3-L3) and upper retainer (U1-U1) were performed at the end of the therapy, and vacuum-formed retainers in both arches were provided for the patient.

The enameloplasty of 13 was necessary at the end of the treatment to reshape it into an upper lateral incisor, to ensure aesthetics and comfort of the patient [18].

### 2.5. Follow-Up and Outcomes

**Figure 6:**
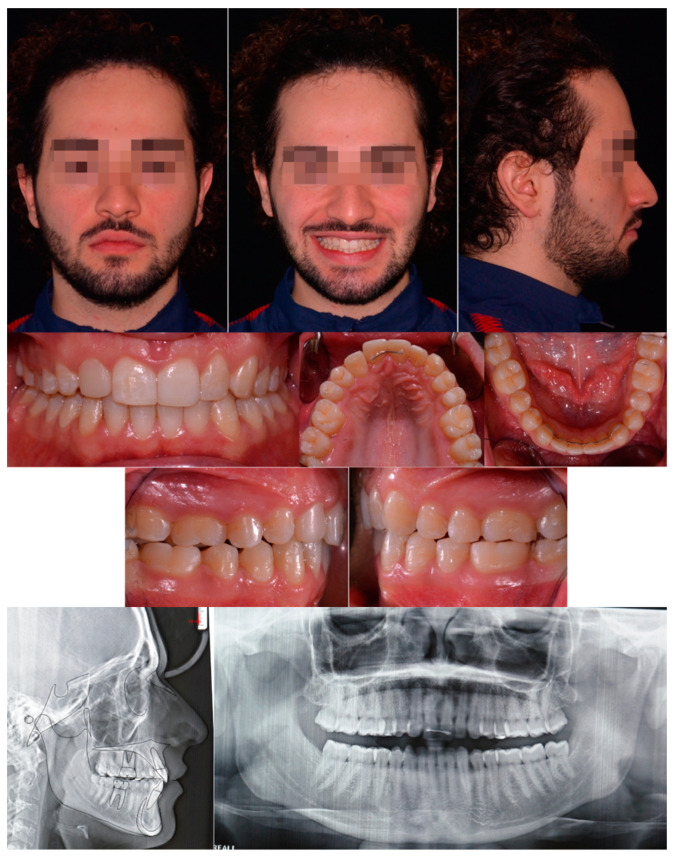
Final extra-, intra-oral, and radiographic records.

The posttreatment records showed that the treatment goals were achieved. Good occlusion and smile aesthetics were reached; moreover, the panoramic X-ray confirmed the body movement of the teeth, with the roots of the teeth parallel to each other. At the end of the therapy, the patient was satisfied with his dental and facial appearance. Final extra-, intra-oral, and radiographic records are all shown in Figure 6 and Table 1.

**Figure 7:**
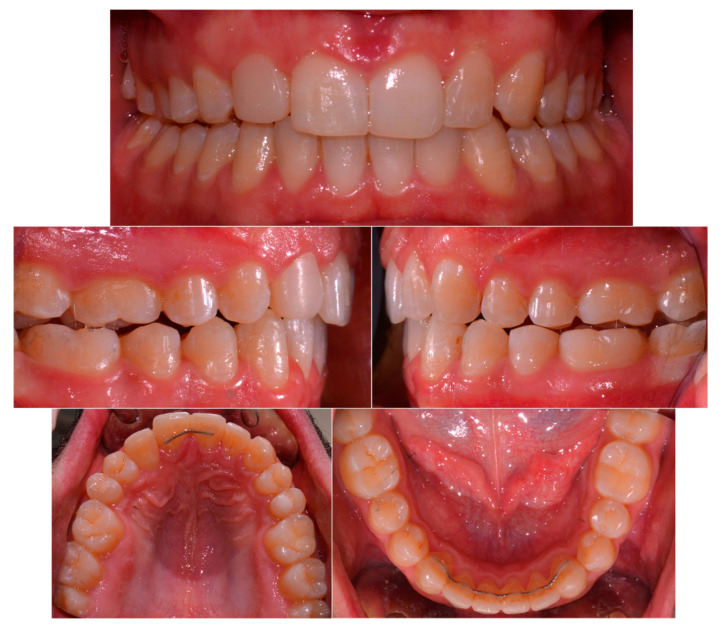
1-year follow-up, intra-oral records.

After 1 year of maintenance, the treatment results were stable (Figure 7). At the end of treatment and 1 year after treatment, the periodontal status of the maxillary canine was normal.

## 3. Discussion

The aim of this study is to present a case of an impacted maxillary canine and agenesis of an upper lateral incisor that was treated with a combined hybrid approach. Due to its location in the aesthetic region, unilateral agenesis of the maxillary lateral incisor may have affected the patient’s quality of life [11]. In these cases, the decision of whether to open or close the space should be individualized for each patient. Patients with agenesis of the lateral incisors who have excessive gingival exposure when they smile, especially younger ones, should not be treated with reopening of the space and dental implant rehabilitation [19]. In fact, the long-term occlusal, gingival, and periodontal outcomes achieved with this approach seem to be worse than space closure [13]. Furthermore, the healthy gingival tissues and the integrity of the interdental gingival papillae will change over a lifetime in harmony with the patient’s own teeth, representing another important advantage of the space closure treatment [20]. With canine root palatal torque increase, differential intrusion of the first premolars, canine extrusion and surgical crown lengthening to follow the micro aesthetics parameters, and bleaching and reshaping of the upper front teeth using ceramic or composite veneers, the space closure approach can produce excellent long-term results [21,22,23].

The use of clear aligners provides several advantages, including aesthetics, comfort, better control of oral hygiene and precise tooth movement compared to traditional fixed orthodontic appliances [1,24,25,26,27].

Due to the patient’s aesthetic request, an orthodontic hybrid approach with clear aligners was preferred, because the use of these devices alone would not be sufficient to move the canine into the dental arch. In fact, the combined use of fixed appliances and clear aligners has simply increased the possibilities with which orthodontists can successfully treat a variety of complex malocclusions [28]. Most orthodontists realized that the hybrid technique could overcome the limitations of the fixed appliances and aligners to achieve more modern and aesthetic orthodontic solutions [29].

In this case, the first problem we met, which cannot be solved by aligners on their own, is surgical exposure and traction of the canine. For this reason, the *Canine First Technique* was used [17]. Indeed, this approach still ensures the aesthetics that are important in adult patients and offers several other advantages. Cantilevers on miniscrews are a determined force system, so all forces and moments can be easily read and calculated [30]. Thus, it is possible to pull the canine to its correct position in the dental arch in a more predictable and safe way, avoiding damages to the roots of the adjacent teeth. Another advantage related to the use of temporary anchorage devices is the elimination of any side effects on the dental elements.

Skeletal anchorage has not only been used for canine traction but also in an indirect approach. In particular, one of the main limitations of aligner treatment is the root torque read-out [2,31]. In this case it was very important to increase the torque of the canine to replicate the torque of the lateral incisor and not to make the canine bulge visible while the patient was smiling. Therefore, to overcome this problem, an activated sectional on the third order and an inverted attachment of a lower premolar was required to make the maximum positive root-palatal torque reading.

The limitation of this study is that it is only a case report. Hence, more scientific literature concerning maxillary impacted canine and incisor agenesis treatment with the use of clear aligners and the *Canine First Technique* is needed to improve and validate this technique. More widely, further research in impacted canines cases treated with a hybrid approach is necessary to learn more about this issue.

## 4. Conclusions

In conclusion, the presented case highlights the effectiveness of an orthodontic treatment with clear aligners in space closure treatment due to agenesis of the lateral incisor with an included canine.

The advantages of clear aligners, including aesthetics, comfort, and improved oral hygiene, could lead to their choice over traditional fixed appliances. The controlled forces exerted by aligners allow for precise tooth movement, enabling the safe and gradual alignment of impacted canines with the dental arch.

## Figures and Tables

**Table 1:** Pre- and posttreatment cephalometric measurements.

Cephalometric Parameter	Pretreatment	Posttreatment
Maxillary Position SNA^	81.6°	82.1°
Mandibular Position SNPg^	78.9°	79.6°
Sagittal Jaw Relation ANPg^	2.7°	2.5°
Maxillary inclination SN^ANS-PNS	10.3°	9.0°
Mandibular inclination SN^GoGn	34.8°	33.8°
Vertical Jaw Relation ANS-PNS^GoGn	24.5°	24.9°
Maxillary Incisor Inclination	105.2°	108.9°
Mandibular Incisor Inclination	89.1°	95.4°
Mandibular incisor compensation	−0.3 mm	−1 mm
Mandibular incisor position	1.2 mm	3.9 mm
Maxillary incisor position	−1.6 mm	3.0 mm
Overjet	4.3 mm	3.9 mm
Overbite	3.1 mm	3.0 mm
Interincisal Angle	141.2°	130.8°
Lower lip protrusion	−4.1 mm	−4.5 mm
Co-Go-Me Angle	125.9°	123.4°

## Data Availability

All data generated or analyzed during this study are included in this article.
